# Influence of intrinsic and extrinsic attributes on neonate survival in an invasive large mammal

**DOI:** 10.1038/s41598-021-90495-x

**Published:** 2021-05-26

**Authors:** Sarah M. Chinn, John C. Kilgo, Mark A. Vukovich, James C. Beasley

**Affiliations:** 1 University of Georgia Savannah River Ecology Laboratory, Aiken, SC 29803 USA; 2 Warnell School of Forestry & Natural Resources, Athens, GA 30602 USA; 3 USDA Forest Service Southern Research Station, New Ellenton, SC 29809 USA; 4 USDA Forest Service-Shawnee National Forest, Vienna, IL 62995 USA

**Keywords:** Population dynamics, Invasive species

## Abstract

Understanding factors influencing survival of neonates for wild species is important for successful management, particularly for determining drivers of population dynamics. Wild pigs (*Sus scrofa*) are invasive and populations are rapidly increasing in part due to high reproductive capacity. Survival of adults is generally high, however, survival of piglets, and particularly neonates, is largely unknown. We located neonates at the natal nest and quantified survival in relation to individual and maternal biological attributes, and environmental variables. During 2017–2020, we captured 50 neonates from 13 litters and documented 28 mortalities (56%) over six weeks. Survival was positively influenced by pelage coloration, likely as a form of camouflage from predators. Male neonates had higher survival. They were born larger than females, which could be beneficial for thermoregulation and competition for milk. Neonates born to larger sows had lower survival. Sow size was positively correlated with litter size, and this finding may reflect the increased nutritional demands of sustaining large litters, or difficulties in defending more neonates against predators. Neonates born in warmer months had higher survival than those born in cooler months. Neonates are inefficient thermoregulators, thus being born in warmer months could be beneficial for maintaining homeostasis as well as access to more food resources. These are the largest and most complete data for neonate wild pig survival and will inform population models for the development of management strategies to reduce negative impacts of this destructive invasive species on native ecosystems.

## Introduction

Population dynamics—how populations change in size and structure over time—is driven by factors such as vital rates (births, deaths), stochastic environmental variation^1^ (e.g., food availability, habitat quality), density dependence^2^ (e.g., predation, disease, immigration, emigration), and demographic variation^2^ (e.g., age structure, sex ratio). Consequently, population dynamics reflect the unique interactions among the environment, physiological and behavioral differences that culminate in individual success to determine the number, spatial distribution, and genetic composition of populations. Understanding drivers that influence populations of wild animals is important for determining why and how populations increase, decrease, and fluctuate spatially and temporally under changing conditions, and thus is vital to inform management and conservation initiatives.

Survival is an integral component of population growth and is associated with fluctuations in a suite of intrinsic biological attributes^3–5^ (e.g., age, genetics, size) and extrinsic environmental factors^6,7^ (e.g., season, landscape characteristics, resource availability). For long-lived vertebrates, juvenile survival is often lower and more variable compared to adults^2,8^, and thus can be one of the most important influences on recruitment. In addition to direct causes of mortality such as predation^9,10^, indirect factors such as body condition and mass at birth, as well as physiological condition have been associated with neonate survival across species^11–14^. Availability of vegetative cover, food, water, and other resources critical to both neonates and the mother are also important for determining behavioral patterns that can influence survival^6,15,16^. Thus, it is imperative to simultaneously assess the effects of individual characteristics, maternal attributes, and environmental factors when studying neonate survival. Elucidating the underlying attributes driving survival is particularly critical in the management of invasive species, which can substantially alter ecosystem-level processes and have extensive economic impacts to agriculture, infrastructure, and human health^17–19^.

Wild pigs (*Sus scrofa*) are among the most widely distributed and damaging invasive species worldwide^20^. Typically comprised of mixed ancestry of wild boar and domestic pigs^21,22^, wild pigs are ecological generalists that are highly adaptable with the greatest reproductive capacity of any mammal of their size, and thus are able to thrive and expand quickly in new environments. Globally, wild pig populations have rapidly increased in abundance and distribution over the past few decades^23^. Thus, understanding attributes that make wild pigs successful within a diversity of landscapes, and the factors that influence population dynamics are necessary to inform management of this invasive species. Although wild pigs are distributed worldwide and have extreme influence over ecosystems across their native and invasive range^24,25^, they remain significantly understudied^26^. Most management plans focus on lethal population control^27^, often neglecting the study of the ecological and biological mechanisms that underlie reasons responsible for their abundance. For example, juvenile survival is lower and more variable compared to adults^28,29^, and plays a prominent role in population dynamics^2^, but is widely ignored in most population models because it is difficult to quantify^30^. Thus, an understanding of factors influencing survival of wild pig neonates is important for successful management, particularly for determining drivers of population dynamics.

While neonate survival assessments for many species of wild ungulates, such as white-tailed deer (*Odocoileus virginianus*), elk (*Cervus elaphus*), bighorn sheep (*Ovis canadensis*), etc., are well-represented in the literature, there are few studies that assess survival for piglets of wild pigs or wild boar^31^ Supplementary Table S1], and none that have successfully quantified neonate survival rates using known-fate approaches^32^. Mortality among domestic piglets ranges between 12–30% and is most precarious during 1–3 days of life, with 50% of all neonate mortality occurring during this period^33^, although it is unknown whether these rates are relevant to wild populations of this species. Baubet et al.^34^ attempted to determine known-fate survival for neonate wild boar within its native range but were unsuccessful due to poor transmitter retention and high rates of abandonment by the sow. Keiter et al.^31^ piloted the use of vaginal implant transmitters (VITs) in invasive wild pigs to determine the location and time of parturition to facilitate capturing and radio-transmitter tagging of neonates at the natal nest. However, their attempt to determine known-fate survival of neonates was unsuccessful due to poor transmitter retention, largely due to transmitter size precluding use on small neonates (~ 1 kg) and removal of the transmitter by the sow or littermates.

Survival of wild pig neonates is likely variable, presumably influenced by a suite of neonate, sow, and environmental attributes. In particular, birth mass is thought to be the most important factor influencing piglet survival among domestic pigs^35,36^. Neonates are born poorly insulated with < 2% body fat, lack brown adipose tissue, and must rely on shivering thermogenesis to maintain adequate body temperature^37,38^. Neonates also have high surface area to volume ratio and must expend energy to maintain internal thermal homeostasis, thus thermoregulation and therefore survival are considered tied to birth mass. Wild pigs exhibit sexual dimorphism^39^ and life-history theory predicts sex-biased mortality from the differential costs and benefits of raising each sex^40^, such that there may be higher maternal energetic requirements for the sex that has a faster growth rate^41^. Mothers in poor condition are predicted to terminate investment in the sex that requires higher energetic cost in the current reproductive event, but at a cost to their own future reproductive success if that sex has greater variance in individual fitness^40^. However, the larger sex may also have intrinsic advantages that increase survival (i.e., larger body size facilitates thermoregulation). Wild pigs also can vary considerably in pelage coloration due to the introgression of domestic genes, which may play a role in camouflage from predators. Another factor that may be important for neonate survival is sow body condition and fat mobilization. Both of these factors are important for lactation^42^, particularly for initiating let down of colostrum, which is necessary for neonates to acquire immunoglobulins^43^. For wild pigs, sow mass is positively associated with age^32,44^ and older sows should have more experience with rearing offspring.

We aimed to provide the first real-time survival monitoring of neonate wild pigs within their invasive range. Our objectives were to assess performance of a very high frequency (VHF) radio-transmitter and quantify neonate wild pig survival to 6 weeks of age in relation to individual biological attributes (mass, pelage color, sex), maternal characteristics (mass), and environmental factors (season). We hypothesized individual attributes would influence survival such that neonates born larger, male, and with the wild-type pelage coloration would have higher probability of survival. We hypothesized neonates born to larger sows would have higher probability of survival. Finally, we hypothesized environmental conditions at the time of birth would influence survival, with neonates born in the warm season having higher probability of survival. Further, among wild species, capture and tagging within the first days of life have been hypothesized to negatively affect neonate survival^45^. Thus, we also aimed to explicitly test if neonates with ear tags had lower survival compared to untagged neonates to determine if tagging itself influenced survival.

## Materials and methods

### Study area

**Figure 1:**
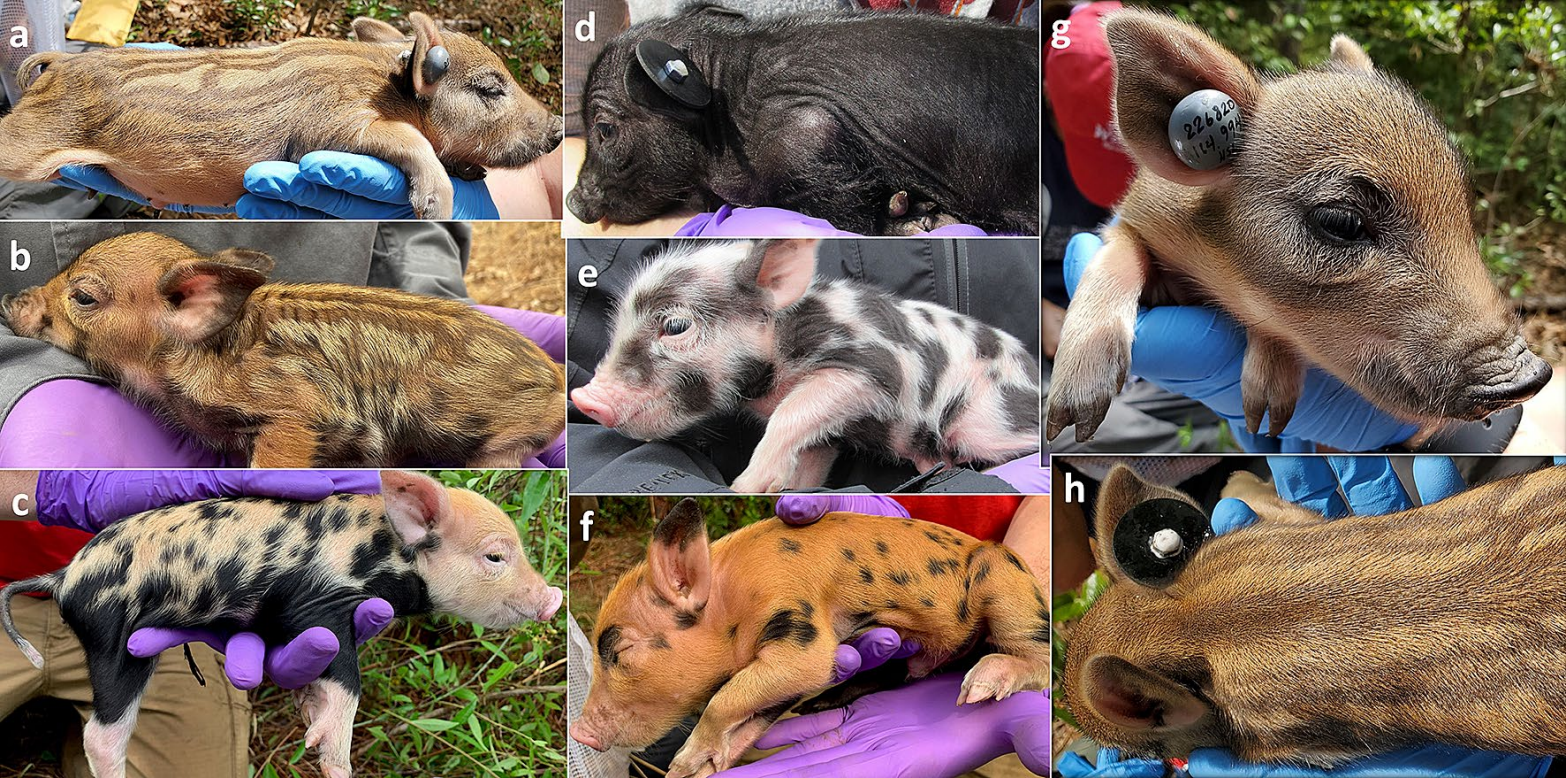
Variation in pelage coloration of wild pig neonates (**a**–**f**) and VHF transmitter ear tag (**g**–**h**). Phenotypic variation in pelage coloration of wild pig (*Sus scrofa*) neonates and VHF transmitter ear tag (Model RI-2BMH; Holohil Systems Ltd.) designed for tracking real-time survival of neonate wild pigs on the Savannah River Site, Aiken, Allendale, and Barnwell counties, South Carolina, USA. (**a**) Wild type, brown striped, (**b**) wild type, brown striped and spotted, (**c**) tri-colored (white, brown and black) spotted, (**d**) solid black, (**e**) black and white spotted, (**f**) red/brown and spotted, (**g**) front view of ear tag (**h**) back view of ear tag.

We conducted our study at the Savannah River Site (SRS) in Aiken, Allendale and Barnwell counties, South Carolina, USA. The SRS is a 80,000-ha U.S. Department of Energy property located in the Upper Coastal Plain physiographic region. The site is primarily composed of a mixture of upland pine habitat and bottomland hardwood swamps and riparian areas. Upland habitat was dominated by loblolly pine (*Pinus taeda*) and longleaf pine (*P. palustris*) that is actively managed for Red-cockaded woodpeckers with prescribed fire. Bottomland hardwood and cypress (*Taxodium distichum*)-tupelo (*Nyssa aquatic* and *N. sylvatica* var. *biflora*) forests characterized the floodplain. The wild pig population on the SRS was descended from feralized domestic pigs released after the private land was converted to a government facility in 1950^44^. Later introduction of wild boar and wild boar and feral pig hybrids led to introgression of wild boar genes into the SRS wild pig population^22,44^. Hybridization on the SRS led to high phenotypic variability, especially in pelage coloration within and between litters (Fig. 1). Despite lethal management, the population has continued to increase over the past several decades to > 5000 individuals^30^. Given the size of the SRS and lack of public access, potential predators of neonate wild pigs (e.g., coyotes, *Canis latrans*; bobcats, *Lynx rufus*) are abundant and widely distributed^10,46^.

### Adult female capture and VIT deployment

All capture and handling of animals was conducted in compliance under approved protocol by the Institutional Animal Care and Use Committee under the University of Georgia (A2015 05-004; A2018 06-024) and the ARRIVE guidelines for the immobilization of animals for studies conducted in the field. From September 2017 to February 2020, we captured adult female wild pigs in corral traps baited with whole corn. We immobilized animals via dart rifle (X-CALIBER, Pneudart, PA) using a combination of Telazol (4.4 mg kg^−1^; MWI Veterinary Supply, ID) and Xylazine hydrochloride (2.2 mg kg^−1^; Wildlife Pharmaceuticals Inc., CO) in 2017–2018, or with a combination of butorphanol (0.43 mg kg^−1^), azaperone (0.36 mg kg^−1^), and medetomidine (0.14 mg kg^−1^) (BAM, 0.0064 ml kg^−1^; Wildlife Pharmaceuticals Inc., CO) and ketamine hydrochloride (2.2 mg kg^−1^; Wildlife Pharmaceuticals Inc., CO) in 2018–2020. Xylazine hydrochloride was antagonized with yohimbine (0.15 mg kg^−1^; MWI Veterinary Supply, ID) and BAM was antagonized with a combination of naltrexone (50 mg ml^−1^; Wildlife Pharmaceuticals Inc., CO) and atipamezole (25 mg ml^−1^; Wildlife Pharmaceuticals Inc., CO), and individuals were monitored until recovery.

Upon immobilization, individuals were weighed, measured, and age was determined through examination of molar eruption patterns^47,48^. We assessed whether captured females were pregnant via serial remote camera images at bait stations, palpation, and a portable ultrasound (SeeMore USB, Interson Corporation, CA). Pregnant females were implanted with a 21 g VIT (M3930; Advanced Telemetry Systems, MN) via methods similar to white-tailed deer studies that previously have been employed for wild pigs^31^. Briefly, the VIT was inserted into the vagina and placed against the cervix with the wings oriented laterally within the body. VITs included a thermistor that sensed a change in temperature upon expulsion during parturition that signaled the number of 30-min intervals elapsed since the change in temperature. Females with VITs were also collared with a VHF (Model TGW-4501; Telonics Inc., AZ) or GPS collar (Model TWG-4577; Telonics Inc. or Vertex Plus; Vectronic Aerospace, Berlin, Germany) to facilitate tracking. Pregnant sows were monitored 3–7 times/week until parturition.

### Neonate capture and handling

Because of the potential of abandonment, we waited 22–48 h after parturition to capture and tag neonates to promote bonding and investment by the sow (e.g., Ref.^34^). We homed to the VIT signal at the farrowing nest, flushed the female, and captured piglets by hand. Once captured, we placed piglets in mesh bags, and subsequently weighed, photographed, and noted sex and pelage coloration of all individuals at the nest location. Within each litter, we tagged a subset of 1–3 piglets with a custom designed VHF transmitter ear tag (Model RI-2BMH; Holohil Systems Ltd., Ontario, Canada). Previous attempts to affix radio transmitters to neonate wild piglets resulted in poor retention or failure^31^. To address these issues, our modified design consisted of a stud ear tag with a nylon post that was approximately 2 cm in diameter and weighed 5 g. We customized it with an internal, coiled antenna to prevent damage or malfunction of the transmitter and included a mortality sensor that activated after 12 h of inactivity. In the field we further modified the tag by using a metal fastener and a small and large neoprene washer on the front and backside of the ear, respectively, to prevent the tag from pulling out (Fig. 1). After these modifications, the tag weighed ~ 10 g at deployment. Piglets were released simultaneously at the natal nest at the conclusion of processing. We deployed remote infrared motion sensor cameras (PC900 HyperFire Professional Series; Reconyx, WI) at the nest area to capture piglet activity post-tagging, document when the female reunited with her offspring, and when the female and piglets left the nest. We also were able to document presence of predators or offspring abandonment by the female through camera images.

### Survival monitoring

We relocated tagged neonates via radio telemetry 3–5 times per week until mortality, tag failure or detachment, or until they reached at least 6 weeks of age. When we detected a mortality signal, we tracked to the location of the transmitter and attempted to determine cause of mortality or whether the tag detached. If there was evidence of mortality, we attempted to determine the cause based upon carcass condition, presence of predator tracks, characteristics of cache sites if any were found, and patterns of carcass consumption^49^. To supplement VHF tracking and allow for tracking of individuals that did not receive VHF tags, we also deployed remote cameras in locations where the sow exhibited localized movements post-parturition. Untagged neonates identifiable from unique pelage coloration patterns that were regularly photographed with remote cameras were included in survival analyses. This method also allowed us to confirm tag detachment versus mortality for any neonate transmitters recovered without adequate evidence of mortality. We assumed mortality occurred on the date the tag was heard in mortality status (12-h delay) if the neonate was not detected with remote cameras. If tagged neonates were not detected during tracking, we homed in to the female signal to confirm the absence of individuals. We attempted this at least twice if the tagged neonate was not detected, thereafter if the transmitter was never recovered and the neonate was not detected on remote cameras, it was assumed deceased either the day after it was last detected or the date it was first not detected (depending on the monitoring schedule). Neonates remain with and are dependent on the sow for the first several months of life^50^.

### Statistical analysis

We used the *lme4* package^51^ in R 3.5.3^52^ to fit linear mixed models (LMM) to estimate if the number of days survived differed between years and tagging status (i.e., whether survival differed for tagged vs untagged neonates), with sow as a random effect since multiple neonates from each sow were monitored. The response variable, days survived, was centered to a mean of 0 and a standard deviation of 1. We checked that model residuals were randomly distributed around zero. If we found no differences in days survived as a function of year or tagging status, we pooled all data for subsequent multivariate survival models.

To evaluate factors that influenced neonate survival, we constructed a series of known-fate models in a Bayesian framework. We included multiple biotic factors such as neonate mass, sex, and pelage color, sow mass, and an environmental attribute (i.e., season). We used sow mass at capture as a proxy for sow condition at parturition and during the six weeks post-parturition. However, changes in mass and body condition could vary between sows between capture and parturition/post-parturition that we were unable to account for that could be important for neonate survival. Seasons (i.e., cold season and warm season) were based on average minimum, maximum, and daily temperature (taken at 15-min intervals) for each month for the timeframe of our study (September 2017–May 2020). Temperature data were recorded in an instrument shelter in the northwest region of the SRS, outfitted with an hygrothermograph (CS-500 Temperature/Relative humidity sensor, Campbell Scientific Inc., UT) to collect minimum and maximum temperature with a minimum accuracy of 1 °C. Minimum accuracy was compared with Vaiasala hand-held probes (Helsinki, Finland) used as reference standards once per year^53^. Cold season was designated as having at least 5 days of minimum temperature ≤ 0 °C and daily average ≤ 12.78 °C (55 °F). The first and second half of March was split into cold and warm season, respectively, because the second half of the month was significantly warmer (12.67 °C and 15.29 °F, respectively; t-test: F_1,91_ = 7.43, *p* = 0.008) and temperatures did not go below freezing in the latter half. We ran our models in Just Another Gibbs Sampler (JAGS^54^) using the *runjags* package^55^ in R. Each model was run for 2,000 iterations with an 8,000 burn in and a thin rate of 1. We checked for model convergence using the Gelman-Rubin diagnostic^56^. We modeled daily survival for each neonate as a series of Bernoulli trials with a probability of success S_*it*_ = P(neonate *i* alive at time *t* | alive at time *t*-1). Daily survival was modeled as logit(S_it_) = x_it_β + Z_γ_, where x_it_ is the covariate, β is the estimate of the covariate, and Z_γ_ is the random effect (in our case, sow). We used a staggered entry modeling approach^57^. Each neonate entered the study on its birthdate (time zero) and its endpoint corresponded to a mortality event or until six weeks old. The first neonates were born in September 2017 and neonates were monitored until May 2020. We chose six weeks because evidence suggests low mortality of wild piglets beyond this time period. A previous study at the same site captured and tagged 71 piglets from 23 litters, ranging in age from neonate (n = 28, tagged 18 neonates) to older than six weeks. All neonate tags failed, however, piglets > 3 kg (approximately six weeks or older) had high tag retention and ~ 94% survival rate (D. Keiter, unpublished data).

We used a conservative approach such that we based our statistical power on the number of events (i.e., mortalities^58^) rather than the number of neonates. Therefore, we only fit models with one to two variables to avoid overfitting. We excluded any neonates from litters that the sow had abandoned, which was ascertained from remote cameras. We accounted for non-independence of samples (e.g., multiple neonates were included from each litter) by nesting the data by sow^59^. Using neonate sex, mass, pelage color (wild, i.e., brown striped, or not wild), sow mass, and season (cold: 1 November-15 March; warm: 16 March-October) we created a candidate set of intrinsic models to evaluate which factors influenced neonate survival. We centered the continuous variables (neonate mass and sow mass) to a mean of 0 and a standard deviation of 1 to allow for standardized comparison. We assessed the continuous variables for correlation using Pearson’s correlation tests, and all pairwise comparisons were r < 0.7, indicating no strong relationships. All parameters were modeled using vague uniform priors.

We used Watanabe–Akaike’s Information Criterion (WAIC), a Bayesian extension of AIC, for model selection to evaluate and rank candidate models^60^. All models within ΔWAICc ≤ 2 of the top model were considered supported. Model weight was used to evaluate the strength of influence among competing models^61^. If a single model did not outperform competing models for best fit to the data, parameters from models with similar WAIC weights were considered influential and reported.

## Results

We deployed 23 VITs across 22 individuals from 2017 to 2020. One female gave birth twice in 2019 (once with a VIT and once without a VIT but we were able to capture piglets through monitoring her movements with GPS data that showed extreme localization indicative of parturition behavior) and once again 2020 (with a VIT; Table 1). Two sows prematurely expelled the VIT (no signs of nesting behavior such as localized movement, nest construction or parturition) and we were unable to subsequently track parturition. Four sows abandoned nests after we tagged neonates (live piglets we found and tagged), and two sows appeared to have given birth (localized GPS data and constructed a nest) but neonates were never located nor were documented on camera and the sows’ movement behavior was not reflective of caring for offspring (i.e., had wide-ranging movements after giving birth). We also were unable to locate one nest or neonates (but subsequently documented that sow with piglets on camera). We were unable to collect neonate data from these nine individuals, therefore, we obtained neonate data from 15 litters from 13 sows (one sow had three reproductive events as mentioned above; Table 1). From the successful litters, we captured 67 neonates (4.67 ± 1.85, average litter size ± SD) and deployed VHF ear tags on 26 individuals. Of these, we were able to successfully track survival of 50 neonates, 24 tagged and 26 untagged, to mortality or at least 6 weeks old (Supplementary Table S1). The apparent survival rate was 44% (i.e., 22 neonates survived to 6 weeks), across the entire study duration. We excluded seven neonates from further analysis because of missing sex or mass data for those individuals. Thus, our sample for analysis assessing factors that influence survival included 43 neonates from 13 litters and 12 sows (Table 1). We determined the survival fates of these 43 neonates and thus did not requiring censoring.

**Table 1:** Data for all wild pig (*Sus scrofa*) sows captured for neonate survival study, n = 24 and number of VIT deployed, n = 23 at the Savannah River Site, Aiken, Allendale, and Barnwell counties, SC, USA from 2017 to 2020. Nest outcome: successful, neonates tagged and sow returned to nest; failed, neonates tagged and sow abandoned nest; dropped VIT, VIT failure and could not determine parturition event; could not locate, sow gave birth but nest was not located. Note: sow P331 gave birth three times during the study.

Reproductive sow data					
Sow	Capture date	Sow age	Sow mass (kg)	Parturition season	Nest outcome
P223	9/19/2017	Subadult	86.18	Warm	Successful
P772	12/28/2017	Yearling	49.90	Cold	Successful
P708	11/22/2017	Adult	74.84	Cold	Failed
P783	3/23/2018	Subadult	74.84	Warm	Failed
P784	3/23/2018	Subadult	88.45	Warm	Successful
P789	8/9/2018	Adult	83.91	Warm	Failed
P758	10/24/2018	Subadult	58.97	Cold	Failed
P777	9/24/2018	Yearling	58.97	–	Dropped VIT
P795	1/3/2019	Subadult	80.15	Cold	Could not locate
P797	1/13/2019	Adult	60.55	–	Dropped VIT
P321	2/23/2019	Yearling	79.38	Warm	Successful
P326	3/11/2019	Subadult	61.23	Warm	Successful
P328	3/20/2019	Adult	83.65	Warm	Successful
P331	3/25/2019	Subadult	65.32	Warm	Successful
P340	4/9/2019	Adult	94.05	Warm	No piglets
P331*	No VIT	Subadult	–	Cold	Successful
P750	12/13/2019	Yearling	47.63	Cold	Successful
P762	12/13/2019	Adult	81.65	Cold	Successful
P749	1/28/2020	Juvenile	33.11	Warm	Successful
P769	2/5/2020	Juvenile	52.16	Cold	Successful^a^
P796	2/5/2020	Juvenile	49.90	Cold	Successful
P354	2/18/2020	Adult	56.25	Cold	Successful
P355	2/18/2020	Adult	71.62	Cold	No piglets
P331^#^	2/21/2020	Adult	81.65	Warm	Successful

Sow age class was determined in the field by molar eruption patterns^47,48^. Age class categories were: Juveniles (< 1 year); Yearlings (1–1.5 years); Subadults (1.5–3 years); Adults (> 3 years).

^a^Litter was successful because offspring were born alive, however, neonates were preyed on prior to tagging (see details below) and data were excluded from survival analysis using JAGS because sex data were missing.

^*^ indicates second birth event.

^#^ indicates third birth event.

For the 50 neonates, survival was not significantly different across years, p = 0.56. Among the 26 untagged neonates, there were 13 mortalities (50% mortality), and among the 24 tagged neonates there were 15 mortalities (62.5% mortality). Of the 28 neonates that died before 6 weeks old, 16 mortalities occurred within 10 days after birth (57.14%). Average time to mortality for these neonates was 14.75 ± 2.13 days (X̅ ± SE). For the neonates that survived to 6 weeks old, 13 were male (59.1%) and 9 were female (40.9%). Survival was not significantly different between tagged and non-tagged neonates (p = 0.81). Because year and tag status had no influence on survival rates, we pooled all neonates for subsequent analysis.

**Figure 2:**
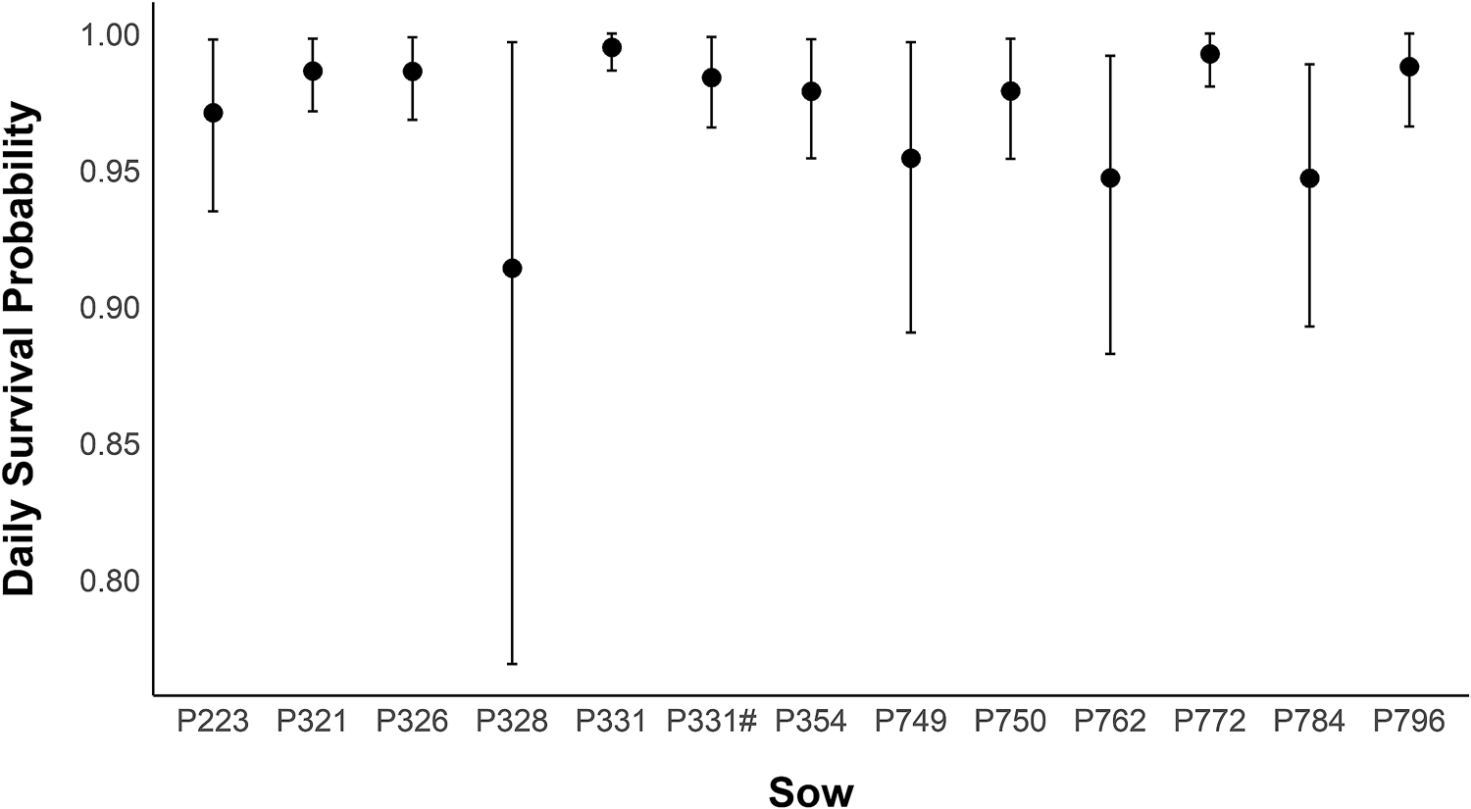
Average daily survival probability of neonate wild pigs. Daily wild pig (*Sus scrofa*) neonate survival probability with 95% CI for each sow on the Savannah River Site, Aiken Allendale, and Barnwell counties, SC, USA from 2017 to 2020. Values are calculated from the null model with sow as a random effect. Note, sow P331 gave birth three times during the study and neonates from two litters were monitored for survival.

**Figure 3:**
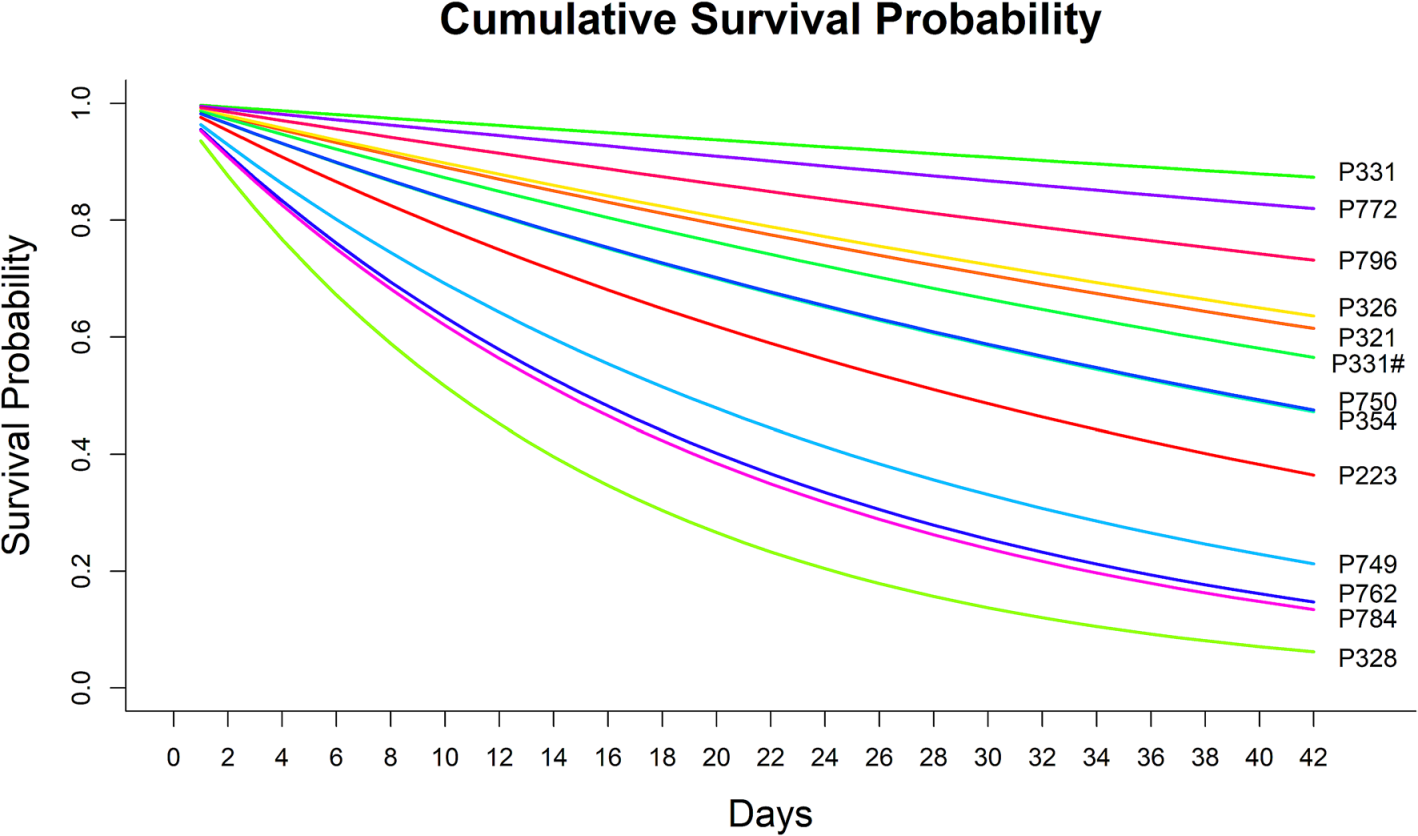
Cumulative survival probability of wild pig neonates to six weeks old. The cumulative survival probability for 13 litters of wild pig (*Sus scrofa*) neonates born to each sow over six weeks at the Savannah River Site, Aiken, Allendale, and Barnwell counties, SC, USA, monitored via radio telemetry and remote camera images, and calculated from the differential daily survival rates for each sow (n = 12; note sow P331 had two litters).

For the 43 neonates for which we had complete data, we found from the null model that only had the intercept and random effect (sow) as parameters, the mean daily survival probability of neonates within each litter ranged from 91.41 to 99.50% (Fig. 2) and cumulative survival probability to six weeks for neonates within each litter ranged from 16.68 to 82.14% (Fig. 3). Our analysis evaluating whether individual, maternal, and environmental attributes influenced neonate mortality resulted in 6 competitive models (Table 2). The top model included only season as being influential to survival. The top model did not carry substantial Watanabe-Akaike weight (*w*_*i*_ = 0.21), so we considered any competing models within Δ2 WAIC (Table 2). The other supported models included neonate sex, sow mass, neonate sex + sow mass, pelage color + sow mass, and pelage color. Neonates born in the colder season had lower survival (β_cold_ = 2.48, 95% credible interval [CI] 1.52–3.00; β_warm_ = 2.56, 95% CI 1.73–3.00) compared to neonates born in the warmer season. Sow mass appeared in three of the top supported models, and as sow mass increased, neonate survival decreased (β_sow mass_ = − 0.77, 95% CI − 3.08 to 1.38). Neonate sex occurred in two of the top supported models, with males having higher survival compared to females (β_male_ = 2.66, 95% CI 2.00–2.76; β_female_ = 2.46, 95% CI 1.61–2.57). Pelage coloration appeared in two of the models; neonates with wild pelage coloration had higher survival (β_wild_ = 2.65, 95% CI 2.05–2.74; β_not wild_ = 2.58, 95% CI 1.87–2.68) compared to other colors (e.g., black and white spotted, solid black; Fig. 1).

**Table 2:** Set of competing models (within ΔWAIC ≤ 2 of the top model) that include neonate, sow and environmental covariates influencing wild pig (*Sus scrofa*) neonate survival to six weeks on the Savannah River Site, Aiken, Allendale, and Barnwell counties, SC USA, 2017–2020.

Model Selection for neonate survival in wild pigs, within ΔAICc ≤ 2			
Model Tested	WAIC	ΔWAIC	Weight
Survival = season	219.80	0.00	0.21
Survival = sex	220.60	0.80	0.14
Survival = sow mass	220.99	1.19	0.11
Survival = sex + sow mass	221.47	1.67	0.09
Survival = wild + sow mass	221.64	1.84	0.08
Survival = wild	221.65	1.85	0.08

Variables included in model selection analyses: litter, litter size; season, season neonate was born (cold = December-15 March or warm = 16 March-September); sex, neonate sex (male or female); sow mass, neonate mass at capture; wild, wild pelage coloration.

While our objective was not to determine cause-specific mortality, we did observe one predation event of a litter (two neonates) prior to tagging. Carcass remains were present at the natal nest site and we observed hemorrhaging, leading us to conclude that the neonates were born alive and were preyed upon. The lack of caching of the remains suggested likely coyote predation. We also observed another event that we could not confidently discern between predation or scavenging of the litter (two neonates) at seven days old, approximately 800 m away from the natal nest. Additionally, we observed one 10-day-old neonate on camera that was emaciated, while the other two littermates appeared healthy. We did not subsequently detect this individual on camera images or with the sow and do not know whether it died from predation or other causes (e.g., abandonment, disease). The cause of the remaining mortalities could not be determined since we were unable to recover the carcasses.

## Discussion

Using a combination of custom-designed VHF ear tag transmitters and remote camera images, here we present the results of the first known fate survival study for neonate invasive wild pigs. Our data suggest survival of neonate wild pigs is relatively high (44%), underscoring the potential for this species to expand in population size and distribution upon establishment in novel environments. Survival for neonate/pre-weaned ungulate species span from 1 to 88%^8^. While wild pig neonate survival falls within the range of other ungulates, unlike other species, wild pigs are physiologically capable of producing multiple litters each year. Indeed, in our study, one sow gave birth to three litters in a span of 14 months (including three gestation periods). However, the extent to which a sow produces multiple litters within a single year across wild pig populations is unknown. Further, wild pigs are able to reproduce at several months of age and produce an average of 5.3 (range 1–12) offspring per litter^32^. These reproductive attributes of wild pigs often exceed those of wild boar due to their descent from feral domestic pigs that were bred for increased reproductive capacity^32,62^, underscoring a critical mechanism by which this species is able to rapidly colonize and expand populations throughout much of their invasive range, even within populations with sustained management efforts^32^.

Parameter estimates in our models generally had overlapping CI’s, likely due to limited sample sizes of neonates and extensive variability among individuals. Nonetheless, our results produced several supported models, suggesting despite generally overall high survival, several biotic and abiotic factors likely contribute to variability in survival of wild pig neonates. As predicted, neonates born in the warm season (n = 23 neonates from six litters) had higher survival compared to those born in colder months (n = 16 from seven litters), when temperatures often fell below freezing in our study area. Temperature is important for neonates because they are smaller compared to adults and lose body heat more quickly to the surrounding environment. Wild pig neonates may be particularly vulnerable to cold temperatures as they are born with little hair and are relatively immobile for the first few days post-parturition. Further, thermoregulation is difficult for neonates, as they are born without brown fat and must expend energy to shiver for thermogenesis^38^. In cold environments neonates lose heat at a rapid rate because of high surface area to volume ratio and temperature difference between thermal homeostasis and the environment. Although the extent to which temperature may serve as a driver of recruitment is not well characterized in the literature, wild pigs occur across much of the globe and can reproduce year round in favorable environments^32^; thus, survival of neonates may be subject to substantial spatial and temporal variability in weather across this species’ range. For example, at the SRS, the primary birthing peak occurs from December–April (with some variation between years) demonstrating that wild pigs in our study do not avoid reproduction during the colder months^44^ (Chinn unpublished data).

While not statistically significant given overlapping CI of the coefficients, likely due to limited sample size, our results also suggest several attributes of individual neonates likely influenced survival. In particular, male neonates had higher survival compared to females. On average, mammalian males are born larger than females^63^, which results in a reduced surface area to volume ratio compared to smaller females. Historically^44^ and in this study, wild pig males were 8.37% larger compared to females (1016.67 ± 37.75 g and 938.16 ± 38.33 g, X̅ ± SE, respectively). Consequently, males may be more efficient in thermoregulation, losing less body heat to the environment, requiring less energy to maintain thermal homeostasis (i.e., less shivering), and are at less risk of chilling and infection^64^. Larger body size could increase male survival, but it is important to note that the sow is in attendance at the nest, potentially providing warmth to the offspring, and the neonates may also huddle together for thermoregulation^37^. In domestic pigs, smaller neonates tend to be the later born offspring in the litter^65^, may take longer to initiate suckling^66^, and consume less colostrum and milk^67^, resulting in lower body condition. The higher birth weight associated with male neonates is associated with increased vigor and survival^68^. However, in our study, neonate mass did not influence survival. Therefore, other traits associated with the male sex, aside from or in combination with mass, are important to survival. Because of sexual dimorphism in wild pigs, male offspring have higher energetic demands^41^ and may outcompete female siblings for high quality teats. Increased size and more energy available (from more milk consumed) could also make male offspring better equipped at traveling and keeping up with the sow when she moves locations. Life-history theory predicts sex-biased offspring mortality, dependent on maternal condition^40^. Poor quality mothers are predicted to terminate investment in the sex that requires higher energetic cost, but at a cost to their future reproductive success if that sex has greater reproductive potential^40^. However, it is not well known if this applies to species with multiple offspring per litter because the size and number of offspring present an investment trade-off^69^. Studies in wild boar found fetal sex ratio (the proportion of males in the litter, i.e., survival of male offspring) was not affected by maternal condition but by litter size^70^ and that sows modulated litter size and sex ratio to increase fitness^71^. Similarly, a study of wild pigs found no relationship between sex ratio and litter size and that sows adjusted litter size as the apparent primary method to increase fitness (Chinn et al. in review). While studies of juvenile wild boar (less than 1 year old) show no difference in survival rates between sexes^72,73^, it is possible that survival may be affected by sex contingent on other factors such as sow quality^40^, environmental condition, population density^72^, or hunting pressure^74^.

Pelage coloration evolved from the needs for concealment from predators, communication, and physiological control and is thus vital for survival^75^. Invasive wild pigs are somewhat unique among mammals as individuals can display extensive variability in phenotype depending on their ancestry. Indeed, piglets within our study area exhibit substantive variability in pelage, and this variability appeared to contribute to differences in survival among individuals. Even within litters individuals may range widely in coat coloration, including black, wild-type, spotted, or other variants in pelage. As predicted, offspring born with wild pelage coloration had higher survival compared to other pelage colors. The wild coloration is characterized by a striped pattern and may include spots (Fig. 1). The stripes break up the solid outline of the neonate and promote blending with the background environment in places with dappled light, promotes crypsis and may decrease detection by predators. This is typical of artiodactyls and species that tend to hide during the first weeks of life^75^. On the SRS, potential predators have high visual acuity and rely primarily on visual cues for hunting^76,77^, and neonates that are not wild patterned may be more conspicuous and at higher risk for predation. Interestingly, the two neonates that were preyed on at the natal nest had non-wild pelage coloration. It is not well understood if pelage coloration has physiological advantages such as thermoregulatory benefits^75^. However, the brown color and black stripes/spots found in the wild pelage coloration could absorb more radiant heat from the environment and aid in conserving body temperature compared to the lighter colored neonates^78^.

In addition to individual characteristics, sow attributes appeared to be an important contributing factor for neonate survival. While not significant, most likely attributable to the limited sample size, neonates born to larger sows generally had lower survival. Larger sows are also associated with larger litter sizes^32^ (Chinn unpublished data) and these larger sows that bear larger litters may have more difficulty defending each neonate from predators. Further, there may be an optimal litter size that corresponds to the largest number of offspring that the sow can successfully provide parental care for (i.e., provide enough food for, keep alive), an extension of Lack’s Principle (which applies to birds^79^) to mammals. This may represent a trade-off where larger sows have more resources (i.e., higher body condition, fat reserves) to produce more offspring, yet these additional neonates have higher probability of mortality. Under optimal conditions, a larger sow may produce many offspring with high survival, thus increasing her reproductive success. A larger sow may bet hedge by producing a large litter and taking advantage of optimal conditions that promote high neonate survival^80^. Similarly, sow mass in wild pigs is positively associated with age^32,44^. Larger and older sows should have more experience rearing offspring and it is intuitive to hypothesize that neonates born to these sows would have higher survival. However, we found the opposite. Larger sows may employ a coin-flipping reproductive strategy by varying the phenotypic plasticity of their offspring^81^. For example, larger sows tend to bear more neonates but may have a mixture of larger and smaller sized neonates or produce neonates with different pelage coloration instead of all the same color. Diversifying phenotypic plasticity of neonates may be advantageous under certain environmental conditions and detrimental in others. Under optimal foraging or environmental conditions, small and larger neonates may both survive, however under limited food conditions or inclement weather, smaller neonates may be more susceptible to mortality. Larger sows with larger litters could have higher neonate mortality if all or a majority of the smaller neonates perish. Depending on the habitat of the nest and where the sow establishes her home range during the first six weeks post-parturition, certain pelage coloration may be more effective camouflage from predators. Larger sows with high variability in neonate pelage color could have higher neonate mortality if the litter is composed of many non-wild pelage color neonates. Conversely, smaller sows are associated with smaller litters and may show more attentiveness over neonates because they are more anxious, thus neonates born to smaller sows may be better defended against predators^82^. These attributes could promote more time spent with neonates, less travel time or distance to resources and decreased risk of inadvertent abandonment during movement.

While survival probabilities for neonates were generally similar among sows, daily survival probabilities for neonates ranged from ~ 91 to 99% among individual sows, and cumulative neonate survival probability to six weeks old ranged from ~ 17 to 82%. Neonates born to four sows (P328, P749, P762, P784) had substantially more variation in daily survival, and the lowest cumulative survival probabilities across the duration of the study period. Thus, even with our small sample size, we demonstrate there is likely some variation in survival of neonates among individual sows that may be influenced by numerous biotic and abiotic factors. There did not appear to be any similar biological attributes among these four sows that we were able to quantify (i.e., age or mass); however, it is possible there were similar traits that we were not able to measure (i.e., foraging efficiency, parental care behavior, social status) that could have contributed to lower survival of their offspring. Resource availability and distribution, as well as predator density within an individual’s home range also likely contributed to variability in neonate survival among sows, which we were unable to quantify for individuals tracked in this study. For example, the resources available during pregnancy and/or lactation may influence behavioral patterns such as time spent foraging and selection of habitat, as well as behavioral trade-offs related to concentrating foraging opportunities in areas with high-quality food resources that may present increased risk of contact with predators of neonates. Increased food intake during gestation has also been correlated with higher birth weight of neonates in domestic pigs^83^.

Capturing and tagging neonates has the potential to decrease their survival^45^. For example, collars or tags with VHF or Global Positioning System (GPS) capabilities may be cumbersome for neonates and may have negative effects on survival or behavior in individuals of any age class^31,34,45^. While we could not control for capture-related impacts in our study, we found that survival of tagged neonates did not differ compared to untagged neonates. Thus, the use of small ear tag transmitters did not appear to have a significant effect on mobility, suckling, or foraging, nor did the tag increase neonate abandonment or rejection by the mother or introduce novel infection/physical disturbance. This suggests use of this style of ear tag may be more effective for tracking neonate movement and survival than alternative models that have been evaluated^31,34^, but further improvements to the design of these transmitters would broaden their utility in studies of ungulate neonates. For example, use of an internal antenna and rubber washers eliminated issues related to transmitter retention experienced by Keiter et al.^31^, but in consequence the range of the VHF signal was severely shortened (~ 100 m). We were able to overcome this limitation through tracking transmitters placed on sows but having to routinely home in within 100 m of neonates could increase the probability of disturbance, and greatly extends the amount of time needed to monitor neonates.

Wild pigs employ a suite of strategies such as high reproductive capacity and generalist foraging behavior to maintain high population numbers^23,62^. Hence, wild pigs are among the most successful large-sized invasive mammals globally. Our data are the first quantification of neonate survival of wild pigs in their invasive range that will inform population models for the development of effective management strategies to ultimately reduce negative impacts of this destructive invasive species on native ecosystems, livestock, and human health. Future research should explore more factors influencing wild pig neonate survival such as resource selection by the sow, neonate genetics, wild pig and predator density in a larger sample as well as in other areas within their invasive range.

## Supplementary Information

Supplementary Information.

## Data Availability

Data will be archived in a digital repository upon acceptance of the manuscript.
