# Supplementary material for: Influence of intrinsic and extrinsic attributes on neonate survival in an invasive large mammal
Source: Sci Rep. 2021 May 26;11:11033. doi: 10.1038/s41598-021-90495-x (PMC8155080; doi:10.1038/s41598-021-90495-x)
Supplement: Supplementary file 1 — Supplementary Information. [file 41598_2021_90495_MOESM1_ESM.pdf]

Supplementary Material

Influence of intrinsic and extrinsic attributes on neonate survival in an invasive large mammal

Sarah M. Chinn[1,2]*, John C. Kilgo[3], Mark A. Vukovich[3,4], James C. Beasley[1,2]

[1]University of Georgia Savannah River Ecology Laboratory, Aiken, SC 29803

[2]Warnell School of Forestry & Natural Resources, Athens, GA 30602

[3]USDA Forest Service Southern Research Station, New Ellenton, SC 29809

[4]USDA Forest Service-Shawnee National Forest, Vienna, IL 62995

* corresponding author: sarahchinn@uga.edu

Table S1. Data for wild pig (*Sus scrofa*) neonates captured for survival study and used in analyses, n = 50 at the Savannah River Site, Aiken, Allendale, and Barnwell counties, SC, USA from 2017–2020. Neonate ID: Sow ID + neonate number in order of processing during capture; Mass: neonate mass at capture (1-3 days old); Pelage Color: wild = brown with black stripes/spots; Litter size: number of live neonates captured at natal nest; VHF Tag: Y = neonate tagged with VHF radio transmitter tag, N = neonate not tagged, tracked by unique pelage coloration with remote camera images; JAGS Analysis: Y = neonate included in Bayesian survival analysis model, N = neonate missing data (sex, mass) and was not included in Bayesian survival analysis model but used for summary statistics and model diagnostics; Days Tracked: number of days neonate survived from birthdate, maximum number of days tracked = 42, mortality event occurred if tracked < 42 days.

Neonate Data

Neonate ID	Sex	Mass (g)	Pelage Color	Litter Size	VHF tag	JAGS analysis	Days Tracked
P223_P1	male	1200	black and white spotted	6	N	Y	24
P223_P2	female	1100	wild	6	N	Y	36
P223_P3	-	-	wild	6	N	N	36
P321_P1	female	810	tri-color spotted	5	Y	Y	42
P321_P2	male	1060	black and white spotted	5	Y	Y	42
P321_P3	female	1000	black and white spotted	5	Y	Y	23
P321_P4	male	1580	black and white spotted	5	N	Y	28
P321_P5	female	910	black and white spotted	5	N	Y	42
P326_P1	male	930	wild	4	Y	Y	16
P326_P2	female	925	wild	4	Y	Y	42
P326_P4	male	870	wild	4	N	Y	42
P328_P1	female	980	wild	2	Y	Y	7
P328_P2	female	1230	red/brown and spotted	2	Y	Y	7
P331_P1	male	1020	tri-color spotted	6	Y	Y	42
P331_P2	female	1020	tri-color spotted	6	Y	Y	42
P331_P3	male	1020	black and white spotted	6	N	Y	42
P331_P4	male	1080	red/brown and spotted	6	N	Y	42
P331_P5	female	700	black and white spotted	6	N	Y	42
P331_P6	male	940	black and white spotted	6	N	Y	42
P331_P1*	-	-	red/brown and spotted	3	N	N	6
P331_P2*	female	-	black and white spotted	3	N	N	8
P331_P3*	female	-	black and white spotted	3	N	N	8
P331_P1#	female	1090	black and white spotted	5	Y	Y	6
P331_P2#	male	1130	black and white spotted	5	Y	Y	6

P331_P3#	female	820	black and white spotted	5	N	Y	42
P331_P4#	female	1100	black and white spotted	5	N	Y	42
P331_P5#	male	830	black and white spotted	5	N	Y	42
P354_P1	male	800	wild	3	Y	Y	42
P354_P2	female	530	wild	3	N	Y	19
P354_P3	female	800	solid black	3	Y	Y	34
P749_P1	male	810	black and white spotted	6	Y	Y	8
P749_P3	male	680	black and white spotted	6	N	Y	22
P750_P14	male	1090	wild	7	N	Y	42
P750_P4	male	930	wild	7	Y	Y	6
P750_P7	male	980	wild	7	N	Y	42
P750_P8	female	820	wild	7	Y	Y	5
P762_P12	male	1070	wild	8	Y	Y	13
P762_P2	female	930	tri-color spotted	8	Y	Y	23
P762_P9	female	960	wild	8	Y	Y	6
P769_P1	-	-	black and white spotted	2	N	N	3
P769_P2	-	-	red/brown and spotted	2	N	N	3
P772_P1	male	900	solid black	3	Y	Y	42
P772_P2	male	1000	solid black	3	N	Y	42
P772_P3	male	1100	solid black	3	Y	Y	42
P784_P1	female	960	black and white spotted	4	Y	Y	4
P784_P2	male	990	wild	4	Y	Y	37
P784_P3	male	1340	wild	4	N	Y	10
P784_P4	male	1050	black and white spotted	4	N	Y	9
P796_P2	female	1140	wild	3	Y	Y	42
P796_P4	female	-	black and white spotted	3	N	N	42

*Second litter for sow P331
#Third litter for sow P331
